# Health research-strengthening and capacity development: Research support system model in an academic healthcare system

**DOI:** 10.5339/qmj.2022.36

**Published:** 2022-08-05

**Authors:** Furqan B Irfan, Rafael I.G.D.J Consunji, Ibrahim A. Janahi, Guillaume Alinier

**Affiliations:** ^1^Department of Neurology and Ophthalmology, College of Osteopathic Medicine, Institute of Global Health, Michigan State University, West Fee Hall, 909 Wilson Road, East Lansing, MI 48824, United States E-mail: GAlinier@hamad.qa; ^2^Hamad Injury Prevention Program, Hamad Trauma Center at Hamad General Hospital, Hamad Medical Corporation, Doha, Qatar; ^3^Department of Pediatric Medicine, Sidra Medicine Education City, Doha, Qatar; ^4^Weill Cornell Medicine–Qatar, Education City, Doha, Qatar; ^5^Hamad Medical Corporation–Ambulance Service, Al Rayyan Road, Doha, Qatar; ^6^School of Health and Social Work, University of Hertfordshire, College Lane, Hatfield, HERTS, AL10 9AB, UK; ^7^Faculty of Health and Life Sciences, Coach Lane Campus, Northumbria University, Newcastle upon Tyne, NE7 7TR, UK

**Keywords:** Capacity building, mentoring, academic health system, knowledge development, support

## Abstract

Introduction: Healthcare research contributes to the well-being of a population; hence, it is important to use the right system to ensure that junior researchers develop the required skills. Current research-strengthening and capacity development programs might lack a research process-based common framework or model leading to variable and suboptimal outcomes. This study aimed to describe the development and evaluation of a model for health research-capacity development at both individual and institutional levels in a Joint Commission International-accredited governmental healthcare organization in Qatar.

Methods: This retrospective observational study evaluated a research support system employed in Qatar for 1 year and constituted of16 stations, each covering a different topic and supported by an experienced faculty member. We recorded how many faculty members were involved and how many people accessed which stations. We developed an outcomes logistic model and obtained feedback about their experience of using the research support system through a short survey.

Results: Twenty-one faculty members supported a total of 77 participants, representing various professions and specialties. The majority of the participants received support on multiple stations, and the most solicited were study design and methodology (n = 45, 58.4%) and research idea (n = 29, 37.7%). The most common type of research that participants required support for was clinical research (n = 65, 84.4%). Moreover, 58.4% of the participants answered the survey, and their responses attested to their perceived benefit of making use of the research support system.

Conclusion: The research support system presented was positively evaluated by participants and promoted networking. Such aspects are favorable to the development of a research culture within an organization and would be a good addition for implementation in universities running healthcare programs and hospitals with residency programs and a large and varied healthcare workforce. This would contribute to the development of health-related research capacity and quality of research outputs in these institutions.

## Introduction

Health research-capacity development (also known as capacity building) is widely accepted as one of the strongest, cost-effective, and sustainable methods of improving the health of the population.^1,2^ The United Nations Development Program described research-capacity development as the process to individually and collectively function sustainably to identify problems, set goals and objectives, create sustainable institutions and organizations, and provide solutions to important national problems.^3,4^

Research capacity-strengthening approaches focus on individual, institutional, and environmental or national levels to build a sustainable national health research system that contributes to global scientific knowledge and the advancement of medicine and health.^2,5^ Research capacity strengthening at an individual level involves research training and human resource development, whereas institutional capacity strengthening includes developing research capacity of institutional departments and programs.^6-8^ The environmental aspect is mostly also applicable at a national level and includes regulations, resources, and incentives related to establishing research programs and conducting research.^2^

At an individual level, many research training programs, scholarships, and fellowships are available for the development of research skills. They range from postgraduate (masters and doctoral) degrees and research fellowships to short- and long-term research courses and research development cooperation programs.^9^ Building a critical mass of researchers and research groups is essential to create and sustain a national academic health system (AHS). These are also advantageous if employed in an interprofessional and multidisciplinary manner, and this is attainable when implemented directly within a healthcare organization.^10^ Hence, research training and development initiatives targeting researchers and scientists form the core of research capacity-strengthening methods.^9^ However, current research-strengthening and capacity development programs lack a research process-based common framework or model leading to variable and suboptimal outcomes. Thus, this study aimed to describe the development and evaluation of the Research Support System (RSS), a model for health research-capacity development at individual and institutional levels in Hamad Medical Corporation (HMC), a governmental healthcare organization in Qatar accredited by the Joint Commission International (JCI) as an Academic Health Center. (11)

## Methods

This retrospective observational study evaluated the RSS at HMC and opened to other partners across the AHS over one year from August 2015 to July 2016. The study population included physicians, residents, fellows, nurses, allied health professionals, trainees, and researchers.

**Figure 1.**
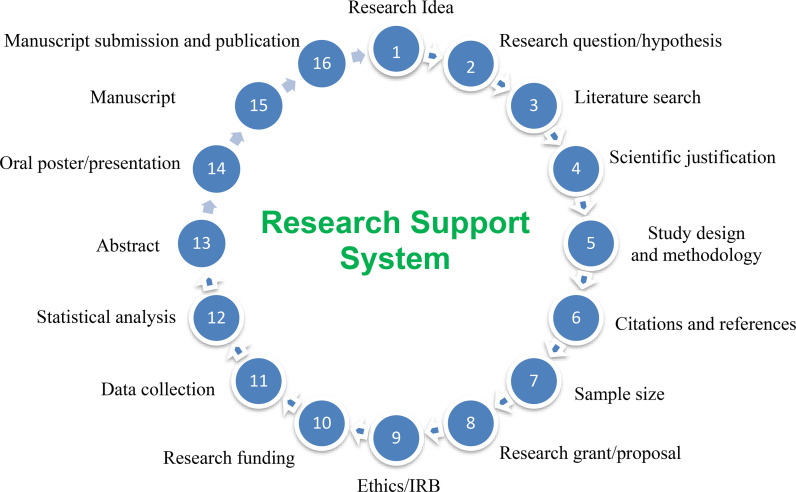
Research support system model

Clinical researchers and basic and molecular scientists, biostatisticians, epidemiologists, clinical academics, and physician scientists working in HMC were approached to form the RSS faculty. The RSS would consist of scientific faculty members who would voluntarily contribute their time and form a human resource pool to provide help, mentorship, and guidance to investigators with their research projects or ideas. The process of a research project was broken down into 16 systematic steps: research idea, research question/hypothesis, literature search, scientific justification, study design and methodology, citations and references, sample size, research and grant proposal, ethics/institutional review board (IRB), funding, data collection, statistical analysis, abstract, oral/poster presentation, writing a manuscript, and paper submission and publication (Figure 1). If an individual was interested to learn all these aspects, the overall format with various stations was similar to that of a formative Objective Structured Clinical Examination (OSCE).^12^

Each RSS faculty member was asked to provide their preference to provide support in one or more of the steps of the research process. Researchers and investigators were invited to bring their research idea/or proposal and have a face-to-face discussion with the RSS faculty member who would help them with the steps of research process. The RSS process engaged the participants with the faculty and advanced each participant's research project in a step-wise manner, all the way from research question and hypothesis to the publication of the paper. The RSS sessions were advertised as “Bring your Research” to well over 20,000 employees via email, websites, posters, and roll-up banners and were held in the afternoon in parallel to the afternoon session of the Research Forum events, which is a biomedicine and health research program where more established researchers present and share their research. Investigators interested to attend were asked to email their queries and research project details to a dedicated RSS email address, and their queries were matched to the particular research process step and the RSS faculty members who would be the most appropriate to provide the required support. The RSS provided support to researchers throughout the year; once they had been put in contact with faculty members during a RSS session, they could continue their interactions when mutually convenient. An outcomes logistic model was utilized for the RSS that included inputs, constraints/barriers, activities, outputs, and outcomes.^8,9^ (Table 1)

The immediate outcome was the number and type of RSS faculty involved and participants who received research support during the study period. Intermediate and long-term outcomes were measured using a short researcher-developed Likert scale survey administered to participants over the phone 1 year after their initial interaction with the RSS faculty. The survey was structured with statements that were rated using a 5-point Likert scale from strongly disagree to strongly agree. The statements were designed to evaluate participants’ experience and perceived benefits with the RSS. It included the following statements: (1) I am satisfied with the research support and guidance received, (2) My understanding and knowledge of research have increased, (3) I have been enabled and encouraged to conduct research, (4) The quality of my research project has been raised, (5) I have networked and shared my research idea and project, (6) I will complete and publish the project with the support received, and (7) I would recommend a colleague to receive support for their research through the research support system. Participants were contacted by telephone after the one-year RSS program and were asked to rate each of the seven statements.

Descriptive analyses were reported as frequencies and percentages for categorical variables. Survey items on participants’ experience and perceived benefits with the RSS were self-reported on a 5-point Likert scale (strongly disagree, disagree, neutral, agree, and strongly agree) and numerically coded as − 2, − 1, 0, 1, and 2, respectively, to calculate the RSS index by taking the mean score of all statements for each participant. Statistical analysis was performed using IBM SPSS Statistics for Windows, version 22.0 (IBM Corp., Armonk, NY <  USA). Student's t-test and one-way analysis of variance were used to obtain significant difference in the mean level of indices between gender and participant job title (nurse, resident/fellow, consultant, and others). A p-value of < 0.05 (two-tailed) was considered significant.

## Results

The volunteer RSS faculty consisted of three professors, three senior biostatisticians, one senior behavioral scientist, one senior basic science and molecular scientist, three post-doctoral basic science and molecular scientists, one PhD-level pharmacist, two physician PhD candidates, and seven physician clinical researchers. They represented a range of professions and specialties, and 17 (81.0 %) were male.

In this study, 77 participants received support and guidance from the RSS in six sessions during the one-year study period. Most participants were male (n = 43, 55.8%). Of the 77 participants, 25 (32.5%) were nurses, 6 (7.8%) were allied health professionals, 5 (6.5%) were pharmacists, 18 (23.4%) were residents and fellows, 19 (24.7%) were physicians, and 4 (5.2%) were researchers. The majority of the participants (n = 42, 54.5%) received support on multiple steps of the RSS from various faculty members. The RSS steps for which most of the participants required support included the following: study design and methodology (n = 45, 58.4%), research idea (n = 29, 37.7%), and research question and hypothesis (n = 17, 22.1%) (Table 1). The participants required support for clinical research (n = 65, 84.4%), genetics and molecular research (n = 5, 6.5%), epidemiological research (n = 4, 5.2%), and translational research (n = 2, 2.5%).

Of the 77 participants who received research support, 45 (58.4%) answered the survey. Moreover, 43 (55.8%) of the respondents were male. The overall mean RSS index score was 1.24 ± 0.94, and the median RSS index score was 1.57 (IQR 0.8–2) (Table 2). The statistical difference in all indices was not significant for participant job title (*p* = 0.55) and gender (*p* = 0.054).

## Discussion

This study describes and evaluated the RSS model for health research-strengthening and capacity development in an academic healthcare system with a relatively nascent research culture. Qatar is developing at a very fast rate with a growing population and healthcare infrastructure. Thus, research is not yet fully ingrained in this relatively young healthcare system, as it has only become an area of interest in the twenty-first century.^13^

The evaluation of the model was determined by assessing the expected outcomes of the logistic model. A total of 21 volunteer RSS faculty members provided research support to 77 participants through the RSS over 1 year. The mean score was >1 and the median score was >2 for all intermediate/long-term outcomes except for whether the quality of participants’ research projects had improved as a result of taking part in the RSS session that had a mean score of 0.98, still corresponding to “agree.” Overall, these results show a reasonably high level of satisfaction with the RSS, even if many of the participants did not yet have a clear research project idea in mind.

More than half of the participants sought out research support on multiple RSS steps, indicating that participants progressed with their research projects. The majority of the investigators received support for “study design and methodology,” which reflects that this is the area most crucial for participants. Frequent support was also received for “research idea” and “research question and hypothesis” since most of the participants were new investigators and wanted to discuss their research ideas and form a hypothesis for their research projects. Thus, other steps that are related to a more advanced stage of conducting a research project, such as writing an abstract, journal publication, grant proposal, or even conference oral or poster presentation were seldom solicited. The RSS was implemented in a public healthcare system that predominantly had full-time clinicians with a burgeoning research interest and from a range of health professions and specialties. Of significance, the vast majority of the healthcare workforce in Qatar is constituted of expatriate professionals who have experienced various educational systems,^13^ rarely including any academic research component. Investigators with genetics, molecular, and translational research interests were also facilitated, thereby assessing the feasibility of the RSS model across the whole research spectrum.

Research-intensive universities have specialized research development centers that help investigators with their research. The overall RSS concept of providing research support by matching an expert faculty with investigators’ research requirements is similar to the functions of the Research Development Core (RDC) at Michigan Institute for Clinical & Health Research (MICHR) and Vanderbilt Institute for Clinical and Translational Research's Studio Program.^14,15^ The RDC at MICHR is focused on grant proposals and pre-award grant support and editing, whereas the Vanderbilt Studio program provides faculty research support for the following seven steps: “hypothesis generation, study design, grant review, implementation, analysis and interpretation, manuscript review, or translation.” ^14,15^ The RSS model differs from the Vanderbilt Studio program in being more specific and holistically broken down into 16 systematic steps that cover all aspects of a research project life cycle (Fig. 1).^15^ The RDC at MICHR, in particular and the Vanderbilt Studio program to some extent, cater to mid-level or established researchers.^14,15^ The generalizability of the RSS model is greater than that of the RDC at MIHCR and the Vanderbilt Studio program, since it is designed to address the needs of established researchers or a new investigator, such as medical students or residents, who are conducting research for the first time. As such, the university-wide implementation of the RSS model could meet the research needs of the faculty members, medical students, residents and fellows, nurses, and other health professionals.

The World Health Organization, United States National Institutes of Health, United Kingdom Department for International Development, and the World Bank have invested and prioritized health research capacity-strengthening initiatives and programs.^16^ Many of such programs specifically aim to address research-capacity development and strengthening: Council on Health Research for Development, Global Forum for Health Research, International Clinical Epidemiology Network, Alliance for Health Policy and Systems Research, Social Science Training and Research Partnership, Training for Health Equity Network, INDEPTH Training and Research Centers of Excellence, Canadian Institute of Health Research, Consortium for Health Policy and Systems Analysis in Africa, African/Asian Regional Capacity Development, and Research on Social Determinants of Health.^17-22^

Mentorship, research training, and hands-on research experience is a key tenet of almost all research-capacity development programs.^11,16,19,23^ According to Brownson et al., such capacity-building approaches can be broadly referred to as “knowledge translation strategies” of which training in its various forms is a key aspect.^24^ It is said to involve leadership, organizational climate and culture, partnerships, workforce development, and financial processes. The authors also stated a reciprocal relationship between how an organization supports the development of its staff and how they shape the organization. Institutional support and partnership with more established universities and institutes also provides opportunities for sharing of knowledge and expertise.^16^ However, the majority of the research-strengthening and capacity development programs that we have found did not have any research process-based framework or model that guides mentorship, training, and institutional support. In the absence of such a model or guidelines, existing barriers and challenges will impede the full potential and deliverable benefits of health research-strengthening programs. These include insufficient time, inadequate funding, not analyzing and interpreting evidence, and lack of cultural and managerial support for research.^24^ Some of the challenges and barriers to health research-strengthening programs that have been described at the interpersonal and institutional levels,^11,16^ can be diminished with the application of a framework like the RSS model. An important opportunity for researchers at all levels is the ability to network within and across institutions, and it was a key aspect of the RSS. The fact that this is employed across professions and specialties was quite unique. This multiprofessional characteristic of the mentors and participants is also a potential advantage, as it helps researchers consider different perspectives and pushes them to carefully describe their project idea so that it can be understood by a different audience.

In the context of a pandemic where physical distancing is required to minimize risks of disease transmission, practices need to be adapted to still offer the required academic support. Teleconferencing platforms have been beneficial among family medicine residents in Qatar in their pursuit of scholarly activity.^25^ The opportunity for more interactive remote activities can generally be created by splitting participants across multiple breakout rooms. Varying language and communication, training and mentoring styles, and expected outcomes^16^ can be streamlined with the incorporation of the RSS model. There is potentially a time-saving element for both faculty and/or participants, as they could access or provide the RSS mentorship from their usual workplace. The faculty could simply carry on with their primary work until a participant contacts them in relation to their allocated step on the RSS model.

### Limitations

The survey response rate to determine intermediate/long-term expected outcomes among RSS participants was 58.4% after the one-year program. The participants were contacted by telephone to answer the survey. Other measures to contact the RSS participants and improve the response rate could have been undertaken (e.g., email). Expected outcomes measured using the logistic model were at the level of investigators. A different survey could have been developed to obtain feedback from the RSS faculty. Long-term outcomes at the institutional level could also have been evaluated. This could have included the effect of RSS on institutional research output, RSS-facilitated number of grants received, RSS-facilitated number of high-quality research publications, and effect of RSS-facilitated research on patient care and outcomes. However, many other confounding factors such as the somewhat transient expatriate workforce and the constantly growing number of publications emerging from HMC over the past couple of decades could also have interfered with the validity of the findings. From a cultural, gender, and professional representation point of view, we noticed an imbalance between the participants and the volunteer RSS faculty, which could also have affected the general experience and feedback given by participants. Contrarily to the overall population in Qatar which includes approximately 75% of male residents, there is a relative parity in the gender distribution among the healthcare professionals, so we could not determine with certainty why the faculty and participants were mostly male. Everyone was equally welcome to share their expertise or attend the support sessions.

## Conclusions

The RSS is a unique health research-strengthening and capacity development model that was implemented in an AHS. Overall, the majority of the participants reported to have had a positive experience and perceived benefits on receiving research support from RSS. Most of the participants required support for study design and methodology, research idea, and research question and hypothesis. RSS provides a research process-based model that promotes networking. We believe that the proposed model was favorable to the development of a research culture within the organization and should be implemented in universities running healthcare programs and hospitals with residency programs and with a large and varied healthcare workforce to strengthen and develop their health-related research capacity and output. We are considering facilitating the RSS as a hybrid activity with an online attendance option to reduce the effect of one of the potential barriers. We would need to determine if the faculty members and participants would find it attractive and convenient in instances when physical attendance is not practical, especially in the interest of saving travel time to the event venue or in case physical distancing needs to be implemented as a public health safety measure.

### Acknowledgments

We would like to express our gratitude to the leadership of the Qatar Academic Health System, especially the HMC Medical Research Center for funding and hosting the Research Forum events during which the RSS sessions were taking place. Special thanks to the RSS faculty for devoting their time to this initiative and sharing their expertise with everyone seeking support.

## Figures and Tables

**Table 1:** Research support system steps for which participants required support.

Research support system process	Number of participants N (%)

Research idea	29 (37.7)

Research question/hypothesis	17 (22.1)

Literature search	4 (5.2)

Scientific justification	4 (5.2)

Study design and methodology	45 (58.4)

Citations and references	4 (5.2)

Sample size	7 (9.1)

Research grant/proposal	9 (11.7)

Ethics/IRB	7 (9.1)

Research funding	3 (3.9)

Data collection	9 (11.7)

Statistical analysis	4 (5.2)

Abstract	1 (1.3)

Oral/poster presentation	2 (2.6)

Write manuscript	2 (2.6)

Manuscript submission and publication	0


**Table 2:** Research support system survey scores.

Research support system (RSS) survey		Survey scores (n=45)	

		Mean ± standard deviation	Median (inter-quartile range)

1	I am satisfied with the research support and guidance received	1.38 ± 1.11	2.0 (1–2)

2	My understanding and knowledge of research have increased	1.27 ± 1.0	2.0 (1–2)

3	I have been enabled and encouraged to conduct research	1.47 ± 0.97	2.0 (1–2)

4	The quality of my research project has been raised	0.98 ± 1.30	1.0 (1–2)

5	I have networked and shared my research idea and project	1.02 ± 1.37	2.0 (0–2)

6	I will complete and publish the project with the support received	1.29 ± 1.08	2.0 (1–2)

7	I would recommend a colleague to receive support for their research through the research support system	1.57 ± 0.95	2.0 (1.3–2)

	RSS index score	1.24 ± 0.94	1.57 (0.8–2)


(5-point Likert scale coded as − 2, − 1, 0, 1, and 2 for strongly disagree to strongly agree, respectively)

